# Impact of metabolic syndrome and its components on heart rate variability during hemodialysis: a cross-sectional study

**DOI:** 10.1186/s12933-016-0328-2

**Published:** 2016-01-27

**Authors:** Yu-Ming Chang, Chih-Chung Shiao, Ya-Ting Huang, I-Ling Chen, Chuan-Lan Yang, Show-Chin Leu, Hung-Li Su, Jsun-Liang Kao, Shih-Ching Tsai, Rong-Na Jhen, Ching-Cherng Uen, On behalf of SMCKD (Saint Mary’s hospital Chronic Kidney Disease) study group

**Affiliations:** Division of Nephrology, Department of Internal Medicine, Saint Mary’s Hospital Luodong, No. 160 Chong-Cheng South Road, Loudong, 265 Yilan Taiwan, ROC; Division of Neurology, Department of Internal Medicine, Saint Mary’s Hospital Luodong, No. 160 Chong-Cheng South Road, Loudong, 265 Yilan Taiwan, ROC; Department of Nursing, Saint Mary’s Hospital Luodong, No. 160 Chong-Cheng South Road, Loudong, 265 Yilan Taiwan, ROC; Saint Mary’s Medicine, Nursing and Management College, No. 100, Ln. 265, Sec. 2, Sanxing Rd., Sanxing Township, Yilan County 266 Taiwan, ROC; Graduate Institute of Clinical Medical Sciences, Chang Gung University, No.259, Wenhua 1st Rd., Guishan Dist., Taoyuan, 33302 Taiwan, ROC

**Keywords:** Autonomic nervous system, Diabetes mellitus, Fasting plasma glucose, Heart rate variability, Hemodialysis, Impaired fasting glucose, Metabolic syndrome

## Abstract

**Background:**

Both uremia and metabolic syndrome (MetS) affect heart rate variability (HRV) which is a risk factor of poor prognoses. The aim of this study was to evaluate the impact of MetS on HRV among chronic hemodialysis patients.

**Methods:**

This cross-sectional study was carried out in a teaching hospital in Northern Taiwan from June to August, 2010. Adult patients on chronic hemodialysis without active medical conditions were enrolled. HRV were measured for 4 times on the index hemodialysis day (HRV-0, -1, -2, and -3 at before, initial, middle, and late phases of hemodialysis, respectively), and the baseline demographic data and clinical parameters during the hemodialysis session were documented. Then we evaluated the impacts of MetS and its five components on HRV.

**Results:**

One hundred and seventy-five patients (100 women, mean age 65.1 ± 12.9 years) were enrolled and included those with MetS (n = 91, 52 %) and without MetS (n = 84, 48 %). The patients with MetS(+) had significantly lower very low frequency, total power, and variance in HRV-0, total power and variance in HRV-2, and variance in HRV-3. (all p ≦ 0.05) When using the individual components of MetS to evaluate the impacts on HRV indices, the fasting plasma glucose (FPG) criterion significantly affected most indices of HRV while other four components including “waist circumference”, “triglycerides”, “blood pressure”, and “high-density lipoprotein” criteria exhibited little impacts on HRV. FPG criterion carried the most powerful influence on cardiac ANS, which was even higher than that of MetS. The HRV of patients with FPG(+) increased initially during the hemodialysis, but turned to decrease dramatically at the late phase of hemodialysis.

**Conclusions:**

The impact of FPG(+) outstood the influence of uremic autonomic dysfunction, and FPG criterion was the most important one among all the components of MetS to influence HRV. These results underscored the importance of interpretation and management for abnormal glucose metabolism.

## Background

Metabolic syndrome (MetS), a clustering of risk factors, is associated with increased risk of developing cardiovascular diseases and diabetes mellitus (DM) [1]. While cardiac autonomic nervous system (ANS) dysfunction has been considered as a complication of MetS and a potential mediator linking MetS and adverse cardiovascular events [2, 3]. Meanwhile, cardiac ANS dysfunction is also found in more than 50 percent of uremic patients treated with maintenance hemodialysis (HD) [4], in whom the autonomic neuropathy is resulted from the defect of baroreceptor, down-regulation of alpha-adrenergic receptors and inappropriately activation of Bezold-Jarisch reflex [5].

Heart rate variability (HRV), which means variation of beat-to-beat interval, is a noninvasive way to evaluate ANS functions. During mild sympathetic stimulation, the HRV indices might increase. However, if the sympathetic stimulation is intense or prolonged, an overall decrease in HRV without correlation with the reduction in sympathetic activity would be seen [6]. While reduced HRV is a significant risk factor for cardiac death, all-cause mortality, development of coronary artery disease and type 2 DM [2, 7, 8]. HRV measurement includes time domain and frequency domain analyses [9, 10]. Among the frequency domain indices, very low frequency (VLF) is thought to be influenced by the thermoregulation of vasomotor tone; low-frequency (LF) activity is widely recognized to reflect a mixture of both the sympathetic and parasympathetic tone; high-frequency (HF) activity has been linked to parasympathetic nervous activity, which is associated with the vagal-medicated modulation of heart rate; LF/HF ratio is an index of sympathovagal balance and thus of autonomic status or sympathetic nervous activities; total power (TP) can be estimated with the sum of the frequencies; whereas variance of the R–R interval values (Var) reflects all the cyclic components responsible for variability in the period of recording [10–15].

Previous studies has investigated the association between MetS and HRV in different participant groups including young adults [16], old adult [17], industrial workers [18], patients with intellectual disabilities [19] or schizophrenia [20], in addition to general population [21]. The results regarding the influence of MetS and the individual components on HRV indices were inconclusive. But generally speaking, the HRV indices tend to decrease in women with MetS comparing to those without MetS, but the changes are inconsistent in men [22].

Nonetheless, the association of MetS and HRV has never been evaluated in chronic HD patients. The association between these two entities might be complicated and different from that in other populations since the uremic autonomic neuropathy per se involves both sympathetic and parasympathetic pathways [23]. Therefore, we conducted current study to elucidate the impact of MetS and its components on HRV at different phases of HD process, which might further evaluate the serial changes of HRV indices under the stress induced by HD in uremic patients.

## Methods

### Ethics, consent and permissions

This cross-sectional study was conducted in a teaching hospital in Northern Taiwan, which was approved by the Institutional Review Board of Saint Mary Hospital Luodong. Written informed consents were obtained from all participants, and the data was analyzed anonymously.

### Study design and populations

Patients were eligible for enrollment if they were adults who underwent maintenance HD with stable conditions during the period from June to August, 2010. Exclusion criteria included patients who were less than 18 years of age, who received HD for less than 3 months, who had arrhythmia or active infection, or who were not willing to receive HRV measurement. Enrolled patients were arranged to receive HRV measurements before HD (HRV-0, as baseline data), and three times during HD (HRV-1, -2, and -3 at initial, middle, and late phases of the index HD session, respectively).

The baseline demographic data, comorbid diseases, etiologies of uremia, and medications were documented from patients’ medical charts. The clinical parameters included blood tests such as complete blood cell count, blood urea nitrogen, serum creatinine, calcium, phosphate, albumin, sodium, potassium, sugar, glycated hemoglobin, intact-parathyroid hormone, and lipid profiles, as well as cardiothoracic ratio were recorded at the time of HRV measurement.

MetS is defined as the presence of any three of the five components: (1) a waist circumference (WC) ≧90 cm (in men) and ≧80 cm (in women); (2) blood pressure (BP) ≧130/85 mmHg or drug treatment for elevated blood pressure; (3) fasting plasma glucose (FPG) ≧100 mg/dl or drug treatment for elevated blood sugar; (4) serum triglycerides (TG) ≧150 mg/dl or drug treatment for elevated triglycerides; (5) serum high-density lipoprotein (HDL) <40 mg/dl (in men) and <50 mg/dl (in women) or drug treatment for low HDL [24]. Other definitions were made as followings: DM, previous usage of insulin or oral hypoglycemic agents; hypertension, usage of anti-hypertension agents or pre-dialysis BP >140/90 mmHg in more than half of the records within the recent 1 month; [25] heart failure, New York Heart Association functional class III or IV.

Then we categorized all participants according to presence or absence of MetS and its five components, and compared the demographic characteristics and the serial HRV indices during HD between (or among) the groups, to evaluate the impacts of MetS and its five components on the individual parameters of HRV before and during HD process.

### Measurements of HRV

HRVs were measured using an analyzer (SSIC, Enjoy Research Inc., Taiwan). It took 5 min while the patients lay quietly with normal breath for more than 20 min. Under a sampling rate of 512 Hz, signals from a lead I electrocardiogram were documented by an 8-bit analog-to-digital converter. Fast Fourier transformation was utilized to perform power spectral analysis which quantified power spectrum into the standard frequency-domain measurements including VLF (0.003–0.04 Hz), LF (0.04–0.15 Hz), HF (0.15–0.40 Hz), TP, LF/HF ratio, and Var [9, 10].

### Statistical analysis

The statistical analyses were performed using the Scientific Package for Social Science (PASW Statistics for Windows, Version 18.0, Chicago: SPSS Inc). Chi square test was used whenever appropriate for comparing categorical variables between two groups. Independent and paired student’s *t* test were performed to evaluate the differences in continuous and non-normally distributed variables between two groups and between different time points during HD in the same group, respectively. Two-way analysis of variance (ANOVA) were performed to evaluate the differences in continuous variables among the four groups (FPG(+)/MetS(+), FPG(+)/MetS(−), FPG(−)/MetS(+), FPG(−)/MetS(−)), while Post Hoc multiple comparison with Bonferroni method for equal variances assumption were further undertaken for group-to-group analysis. Microsoft Office Excel 2013 was used to draw the plots comparing the serial HRV indices among groups. Continuous data were expressed as mean ± standard deviation, whereas categorical variables were shown as number (percentage) unless otherwise specified. In all statistical analyses, two-sided p ≦ 0.05 was considered statistically significant.

## Results

During the study period from June to August, 2010, 202 patients who underwent HD for more than 3 months were screened. After excluding 7 patients with infectious disease, 14 patients with obvious arrhythmia, and 6 patients who hesitated to receive HRV measurement, a total of 175 patients (100 women, mean age 65.1 ± 12.9 years) were enrolled. According to the definitions of MetS and its components, 91 (52.0 %) patients were diagnosed with MetS (MetS(+)), while 79 (45.1 %) patients were WC(+), 128 (73.1 %) were BP(+), 65 (37.1 %) were FPG(+), 63 (36.0 %) were TG(+), and 125 (71.4 %) were HDL(+). As to the associations between MetS and its five components, the diagnosis of MetS was established in 78.5 % of patients with WC(+), 52.3 % of patients with BP(+), 83.1 % of patients with FPG(+), 87.3 % of patients with TG(+), and 69.6 % of patients with HDL(+).

### Comparisons of demographic data between participants with and without MetS

The clinical characteristics of all participants, along with MetS(+) and MetS(−) groups were shown in Table 1. The most frequent cause of uremia in MetS(+) and MetS(−) groups were diabetic nephropathy (51.6 %) and chronic glomerulonephritis (67.9 %), respectively. Comparing with the MetS(−) group, those in MetS(+) group had significantly higher portion of DM (51.6 % versus 9.5 %, p < 0.001), higher WC (90.1 versus 81.1 cm, p < 0.001), along with higher serum TG (208.0 versus 103.5 mg/dL, p < 0.001) and LDL (105.2 versus 90.5 mg/dL, p = 0.001) levels. The MetS(+) group also had lower HDL (25.0 versus 47.2 mg/dL, p < 0.001) and intact-parathyroid hormone (i-PTH, 204.7 versus 373.1 μg/L, p = 0.025). As to the HD-associated parameters, the MetS(+) group had worse dialysis clearance (Kt/V, 1.37 versus 1.50, p < 0.001), but higher dry weight (63.5 versus 51.4 kg, p = 0.007) and baseline BP including systolic BP-0 (134.2 versus 123.4 mmHg, p = 0.006), and mean arterial pressure (93.1 versus 87.7 mmHg, p = 0.036). Other demographic and clinical parameters were not statistically different between the two groups (Table 1).

**Table 1:** Comparisons of demographic data between participants with and without metabolic syndrome

	Total (n = 175)	MetS (+) (n = 91)	MetS (−) (n = 84)	P-value
Age, years	65.1 ± 12.9	65.6 ± 12.3	64.6 ± 13.5	0.600
Gender, woman	100 (57.1 %)	52 (57.1 %)	48 (57.1 %)	1.000
Comorbidities				
Diabetes mellitus	55 (31.4 %)	47 (51.6 %)	8 (9.5 %)	<0.001
Hypertension	128 (73.1 %)	67 (73.6 %)	61 (72.6 %)	0.881
Taking Beta-blockers or ACEi/ARB	56 (32.0 %)	30 (33.0 %)	26 (31.0 %)	0.775
Hypotension	32 (18.3 %)	13 (14.3 %)	19 (22.6 %)	0.154
Taking midodrine	16 (9.1 %)	8 (8.8 %)	8 (9.5 %)	0.867
Heart failure	43 (24.6 %)	22 (24.2 %)	21 (25.0 %)	0.899
Coronary artery disease	43 (24.6 %)	27 (29.7 %)	16 (19.0 %)	0.103
Cerebrovascular disease	23 (13.1 %)	13 (14.3 %)	10 (11.9 %)	0.641
Peripheral arterial disease	13 (7.4 %)	5 (5.5 %)	8 (9.5 %)	0.310
Causes of uremia				<0.001
Diabetic nephropathy	55 (31.4 %)	47 (51.6 %)	8 (9.5 %)	
Hypertension	2 (1.1 %)	1 (1.1 %)	1 (1.2 %)	
Chronic GN	92 (52.6 %)	35 (38.5 %)	57 (67.9 %)	
PCKD	11 (6.3 %)	5 (5.5 %)	6 (7.1 %)	
Chronic IN	4 (2.3 %)	1 (1.1 %)	3 (3.6 %)	
Others	11 (6.3 %)	2 (2.2 %)	9 (10.7 %)	
Baseline data				
Waist Circumference, cm	86.1 ± 11.0	90.1 ± 11.2	81.1 ± 8.6	<0.001
Cardio-Thoracic Ratio, %	0.52 ± 0.05	0.52 ± 0.05	0.52 ± 0.05	0.611
Blood Urea Nitrogen, mg/dL	74.7 ± 20.1	73.9 ± 19.5	75.6 ± 20.7	0.578
Creatinine, mg/dL	10.5 ± 3.5	10.9 ± 4.3	10.2 ± 2.2	0.163
Kt/V	1.43 ± 0.24	1.37 ± 0.23	1.50 ± 0.23	<0.001
Urea Reduction Ratio, %	78.4 ± 54.8	81.5 ± 75.4	75.1 ± 10.1	0.441
Calcium, mg/dL	9.1 ± 0.7	9.1 ± 0.7	9.0 ± 0.7	0.851
Phosphate, mg/dL	4.9 ± 1.7	5.1 ± 1.6	4.7 ± 1.8	0.155
Calcium × Phosphate, (mg/dL)^2^	44.6 ± 15.7	46.1 ± 14.3	43.0 ± 16.9	0.192
Albumin, g/dL	3.8 ± 0.3	3.8 ± 0.3	3.7 ± 0.3	0.097
Potassium, mEq/L	4.7 ± 0.8	4.8 ± 0.8	4.6 ± 0.7	0.159
i-PTH, ug/L	285.5 ± 481.1	204.7 ± 242.9	373.1 ± 637.5	0.025
Hemoglobin, g/dL	9.7 ± 1.4	9.7 ± 1.4	9.7 ± 1.4	0.793
Hematocrit, %	30.2 ± 4.2	30.0 ± 4.1	30.4 ± 4.3	0.492
White blood cell, ×10^9^/L	6.3 ± 2.1	6.4 ± 1.9	6.2 ± 2.4	0.532
Total cholesterol, mg/dL	163.0 ± 35.5	167.8 ± 36.1	157.9 ± 34.3	0.066
Triglyceride, mg/dL	157.8 ± 132.0	208.0 ± 142.0	103.5 ± 94.5	<0.001
Low-density lipoprotein, mg/dL	98.1 ± 30.3	105.2 ± 30.5	90.5 ± 28.3	0.001
High-density lipoprotein, mg/dL	35.6 ± 18.7	25.0 ± 11.6	47.2 ± 18.1	<0.001
Sugar (postprandial), mg/dL	148.2 ± 54.9	160.5 ± 65.5	134.9 ± 36.2	0.001
Glycated hemoglobin, %	7.1 ± 1.5	7.2 ± 1.5	6.8 ± 1.5	0.331
At the index HD				
Dry weight, kg	57.6 ± 29.6	63.5 ± 39.6	51.4 ± 7.9	0.007
Actual UF, kg	2.22 ± 0.94	2.34 ± 0.92	2.10 ± 0.96	0.083
%UF, %	4.02 ± 1.65	3.97 ± 1.55	4.07 ± 1.76	0.680
MAP-0, mmHg (MAP-1)	90.5 ± 17.0	93.1 ± 16.9	87.7 ± 16.7	0.036
SBP-0, mmHg (SBP-1)	129.0 ± 26.1	134.2 ± 23.6	123.4 ± 27.7	0.006
DBP-0, mmHg (DBP-1)	71.7 ± 13.0	73.4 ± 13.2	70.0 ± 12.6	0.081
MAP-1, mmHg (MAP-2)	89.9 ± 17.2	91.3 ± 17.6	88.3 ± 16.8	0.255
SBP-1, mmHg (SBP-2)	125.8 ± 28.6	128.2 ± 27.6	123.2 ± 29.6	0.254
DBP-1, mmHg (DBP-2)	70.8 ± 15.9	72.2 ± 16.0	69.3 ± 15.7	0.237
HR-1,/min (HR-1)	74.1 ± 6.5	74.1 ± 6.2	74.1 ± 6.9	0.990
MAP-2, mmHg (MAP-3)	87.5 ± 16.6	87.4 ± 17.5	87.8 ± 15.6	0.870
SBP-2, mmHg (SBP-3)	123.1 ± 26.1	122.4 ± 27.8	123.9 ± 24.2	0.703
DBP-2, mmHg (DBP-3)	70.2 ± 12.4	70.7 ± 12.4	69.7 ± 12.5	0.628
HR-2,/min (HR-2)	75.2 ± 8.2	74.5 ± 7.7	75.9 ± 8.7	0.625
MAP-3, mmHg (MAP-4)	88.5 ± 16.3	89.0 ± 17.5	88.0 ± 14.9	0.706
SBP-3, mmHg (SBP-4)	125.7 ± 24.5	126.3 ± 24.7	125.0 ± 24.5	0.714
DBP-3, mmHg (DBP-4)	70.7 ± 11.8	71.8 ± 11.7	69.5 ± 12.0	0.213
HR-3,/min (HR-3)	75.6 ± 8.2	75.4 ± 7.4	75.9 ± 9.1	0.728

Values are presented as mean ± standard deviation or number (%) unless otherwise stated. P-value was calculated using Chi square test and independent student’s *t*-test. Baseline laboratory data were the pre-dialysis data obtained when patients receiving HRV measurement

*ACEi* angiotensin converting enzyme inhibitor, *ARB* angiotensin receptor blocker, *CCB* calcium-channel blocker, *DBP* diastolic blood pressure, *GN* glomerulonephritis, *IN* interstitial nephritis, *i-PTH* intact-parathyroid hormone, *MAP* mean arterial pressure, *PCKD* polycystic kidney disease, *SBP* systolic blood pressure, *UF* ultrafiltration, *%UF* ultrafiltration divided by body weight

### Impact on HRV: from MetS and its five components

The impacts of MetS and its five components on the HRV indices at different phases of HD were summarized in Tables 2 and 3. We found that the patients with MetS(+) had significantly lower VLF-0 (4.16 ± 1.79 versus 4.88 ± 1.53, p = 0.027), TP-0 (4.16 ± 1.79 versus 5.77 ± 1.72, p = 0.033), Var-0 (5.24 ± 1.70 versus 5.94 ± 1.62, p = 0.031), TP-2 (5.72 ± 1.85 versus 6.27 ± 1.71, p = 0.043), Var-2 (5.81 ± 1.78 versus 6.38 ± 1.61, p = 0.030), and Var-3 (5.73 ± 1.87 versus 6.31 ± 1.75, p = 0.037).

**Table 2:** Comparisons of the impacts on heart rate variability indices from metabolic syndrome and its five components

	MetS [(+) n = 91] vs [(−) n = 84]	Components of MetS				
		WC [(+) n = 79] vs [(−) n = 96]	BP [(+) n = 128] vs [(−) n = 47]	FPG [(+) n = 65] vs [(−) n = 110]	TG [(+) n = 63] vs [(−) n = 112]	HDL [(+) n = 125] vs [(−) n = 50]
HRV-0						
VLF-0	↓#	↓#	NS	↓#	↓#	NS
LF-0	NS	NS	NS	↓#	NS	NS
HF-0	NS	NS	NS	↓#	NS	NS
LF/HF-0	NS	NS	NS	NS	NS	NS
TP-0	↓#	NS	NS	↓#	NS	NS
Var-0	↓#	NS	NS	↓#	↓#	NS
HRV-1						
VLF-1	NS	NS	NS	↓#	NS	NS
LF-1	NS	NS	NS	NS	NS	NS
HF-1	NS	NS	NS	NS	NS	NS
LF/HF-1	NS	NS	NS	NS	NS	NS
TP-1	NS	NS	NS	↓#	NS	NS
Var-1	NS	NS	NS	↓#	NS	NS
HRV-2						
VLF-2	NS	NS	NS	↓#	NS	NS
LF-2	NS	NS	NS	NS	NS	NS
HF-2	NS	NS	NS	NS	NS	NS
LF/HF-2	NS	NS	NS	NS	NS	NS
TP-2	↓#	NS	NS	↓#	NS	NS
Var-2	↓#	NS	NS	↓#	NS	NS
HRV-3						
VLF-3	NS	↓#	NS	↓##	NS	NS
LF-3	NS	NS	NS	↓#	NS	NS
HF-3	NS	NS	NS	↓#	NS	NS
LF/HF-3	NS	NS	NS	↓#	NS	NS
TP-3	NS	NS	NS	↓##	NS	NS
Var-3	**↓#**	NS	NS	↓##	NS	NS

Values are presented as mean. p-value was calculated using independent student’s *t*-test. HRV-0, -1, -2, and -3 were HRV measured before HD, and at initial, middle, and late phases of the index hemodialysis session, respectively

↑ and ↓ denote higher and lower value in participants met the criterion comparing with those didn’t meet the criterion. ^#^ p ≦ 0.05, ^##^ p ≦ 0.001 in the comparison. The data with significant differences were expressed as mean mean ± standard deviation

Units: Ln(ms^2^) in VLF, LF, HF, TP, and Var; Ln(ratio) in LF/HF ratio

*BP* blood pressure, *FPG* fasting plasma glucose, *HDL* high-density lipoprotein, *HF* high frequency, *HRV* heart rate variability, *LF* low frequency, *MetS* metabolic syndrome, *NS* not significant, *TG* triglycerides, *TP* total power, *Var* variance of the R–R intervals, *VLF* very low frequency, *WC* waist circumference

**Table 3:** Comparisons of the heart rate variability indices of the groups

	Total (n = 175)	Comparisons between two groups [FPG(+) versus FPG(-)]			Comparisons among four groups [FPG(+)/MetS(+), FPG(+)/MetS(−), FPG(−)/MetS(+), and FPG(−)/MetS(−)]	
		FPG(+) (n = 65)	FPG(−) (n = 110)	P-value	Intergroup analysis, p-value	Post Hoc analysis using Bonferroni method, p-value
HRV-0						
VLF-0	4.53 ± 1.69	4.07 ± 1.77	4.80 ± 1.60	0.031		
LF-0	3.24 ± 3.33	2.21 ± 4.73	3.85 ± 1.91	0.013		
HF-0	3.03 ± 3.60	2.08 ± 5.26	3.59 ± 1.92	0.035		
LF/HF-0	0.21 ± 1.20	0.13 ± 1.29	0.26 ± 1.14	0.577		
TP-0	5.41 ± 1.80	4.87 ± 2.03	5.72 ± 1.58	0.017	0.046	NS
Var-0	5.60 ± 1.69	5.16 ± 1.90	5.86 ± 1.51	0.037		
HRV-1						
VLF-1	4.94 ± 1.74	4.59 ± 1.75	5.15 ± 1.70	0.041		
LF-1	3.49 ± 3.62	3.08 ± 2.24	3.75 ± 4.23	0.238		
HF-1	3.19 ± 3.88	2.92 ± 1.90	3.35 ± 4.68	0.483		
LF/HF-1	0.31 ± 1.21	0.15 ± 1.16	0.40 ± 1.23	0.196		
TP-1	5.67 ± 1.82	5.25 ± 1.76	5.93 ± 1.82	0.015		
Var-1	5.81 ± 1.69	5.38 ± 1.64	6.07 ± 1.67	0.009	0.045	NS
HRV-2						
VLF-2	5.24 ± 1.67	4.77 ± 1.56	5.52 ± 1.68	0.004	0.032	(+/+) vs (−/−), p = 0.030
LF-2	3.88 ± 3.64	3.37 ± 2.15	4.18 ± 4.27	0.158		
HF-2	3.45 ± 3.93	3.14 ± 2.07	3.63 ± 4.71	0.430		
LF/HF-2	0.43 ± 1.08	0.24 ± 1.03	0.55 ± 1.09	0.060		
TP-2	5.98 ± 1.80	5.47 ± 1.67	6.28 ± 1.80	0.004	0.031	(+/+) vs (−/−), p = 0.024
Var-2	6.08 ± 1.72	5.56 ± 1.72	6.39 ± 1.65	0.002	0.017	(+/+) vs (−/−), p = 0.014
HRV-3						
VLF-3	5.05 ± 1.95	4.29 ± 2.04	5.49 ± 1.75	<0.001	0.001	(+/+) vs (−/−), p = 0.005 (+/+) vs (−/+), p = 0.001
LF-3	3.63 ± 4.30	2.29 ± 5.28	4.43 ± 3.37	0.001	0.011	(+/+) vs (−/−), p = 0.034 (+/+) vs (+/−), p = 0.020
HF-3	3.15 ± 4.69	2.03 ± 5.84	3.82 ± 3.72	0.015		
LF/HF-3	0.48 ± 1.10	0.26 ± 1.24	0.61 ± 0.99	0.040	0.049	NS
TP-3	5.83 ± 2.04	5.02 ± 2.18	6.31 ± 1.80	<0.001	0.001	(+/+) vs (−/−), p = 0.002 (+/+) vs (−/+), p = 0.002
Var-3	6.00 ± 1.84	5.23 ± 1.90	6.47 ± 1.64	<0.001	<0.001	(+/+) vs (−/−), p = 0.001 (+/+) vs (−/+), p = 0.002

Values are presented as mean ± standard deviation. Independent student’s *t*-test and two-way analysis of variance (ANOVA) were used to perform comparisons between two groups (FPG(+) versus FPG(-)) and among four groups (FPG(+)/(-) subgrouped by MetS(+)/(-)), respectively

HRV-0, -1, -2, and -3 were HRV measured before HD, and at initial, middle, and late phases of the index hemodialysis session, respectively

Units: Ln(ms^2^) in Var, TP, VLF, LF, and HF; Ln(ratio) in LF/HF

*FPG* fasting plasma glucose, *HRV* heart rate variability, *LF* low frequency, *MetS* metabolic syndrome, *NS* not significant, *TP* total power, *Var* variance of the R–R intervals, *VLF* very low frequency

When using the individual components of MetS to evaluate their impacts on HRV indices, the four components including WC, TG, HDL and BP exhibited little impacts on HRV. WC(+) is only associated with lower VLF-0 (4.22 ± 1.75 versus 4.98 ± 1.47, p = 0.025) and VLF-3 (4.74 ± 2.16 versus 5.58 ± 1.53, p = 0.008). TG(+) is related to decreased VLF-0 (3.96 ± 1.84 versus 4.83 ± 1.54, p = 0.011) and Var-0 (5.16 ± 1.82 versus 5.83 ± 1.58, p = 0.048). Whereas both HDL(+) and BP(+) did not contribute to differences in HRV indices (Table 2).

However, the patients with FPG(+) were associated with significantly lower HRV indices including most indices of HRV. The comparisons of the HRV indices between FPG(+) and FPG(−) groups were shown in Table 3. The HRV indices with statistical significances included VLF-0, LF-0, HF-0, TP-0, Var-0, VLF-1, TP-1, Var-1, VLF-2, TP-2, Var-2, VLF-3, LF-3, HF-3, LF/HF ratio-3, TP-3, and Var-3 (Table 3).

### Impact on HRV: from FPG criterion

As mentioned above, the patients with FPG(+) had significantly lower values of VLF, TP, and Var through HRV-0 to HRV-3, along with lower values of LF and HF at both HRV-0 and HRV-3, and lower LF/HF ratio at HRV-3 (Tables 2, 3).

In the FPG(−) group, almost all HRV indices (LF, HF, TP, Var, and LF/HF ratio) continuously increased during the HD process (all p ≦ 0.001 when HRV-1 comparing with HRV-2, and HRV-2 comparing with HRV-3). As to the rest indice, VLF, its value increased from HRV-1 to HRV-2, but turned to decrease a little from HRV-2 to HRV-3 (both p ≦ 0.001). On the contrary, almost all HRV indices (VLF, LF, HF, TP, and Var) in the FPG(+) group increased initially during the HD process (from HRV-1 to HRV-2, all p ≦ 0.001), but turned to decrease dramatically at the late phase of HD to the levels which were lower than the levels at HRV-2 (from HRV-2 to HRV-3, all p ≦ 0.01). Whereas the values of LF/HF ratio persistently increased from HRV-1 to HRV-3 (both p values ≦0.01). (the raw data regarding the comparisons of HRV in different phases of HD were not shown). As a result, the differences between the FPG(−) and FPG(+) groups of most HRV indices became larger gradually as the HD processed, and all the HRV indices were significantly different between FPG(+) and FPG(−) groups at the late phase of HD (HRV-3). (all p ≦ 0.001 in VLF, LF, TP, and Var; and ≦0.05 in HF and LF/HF ratio).

**Fig. 1:**
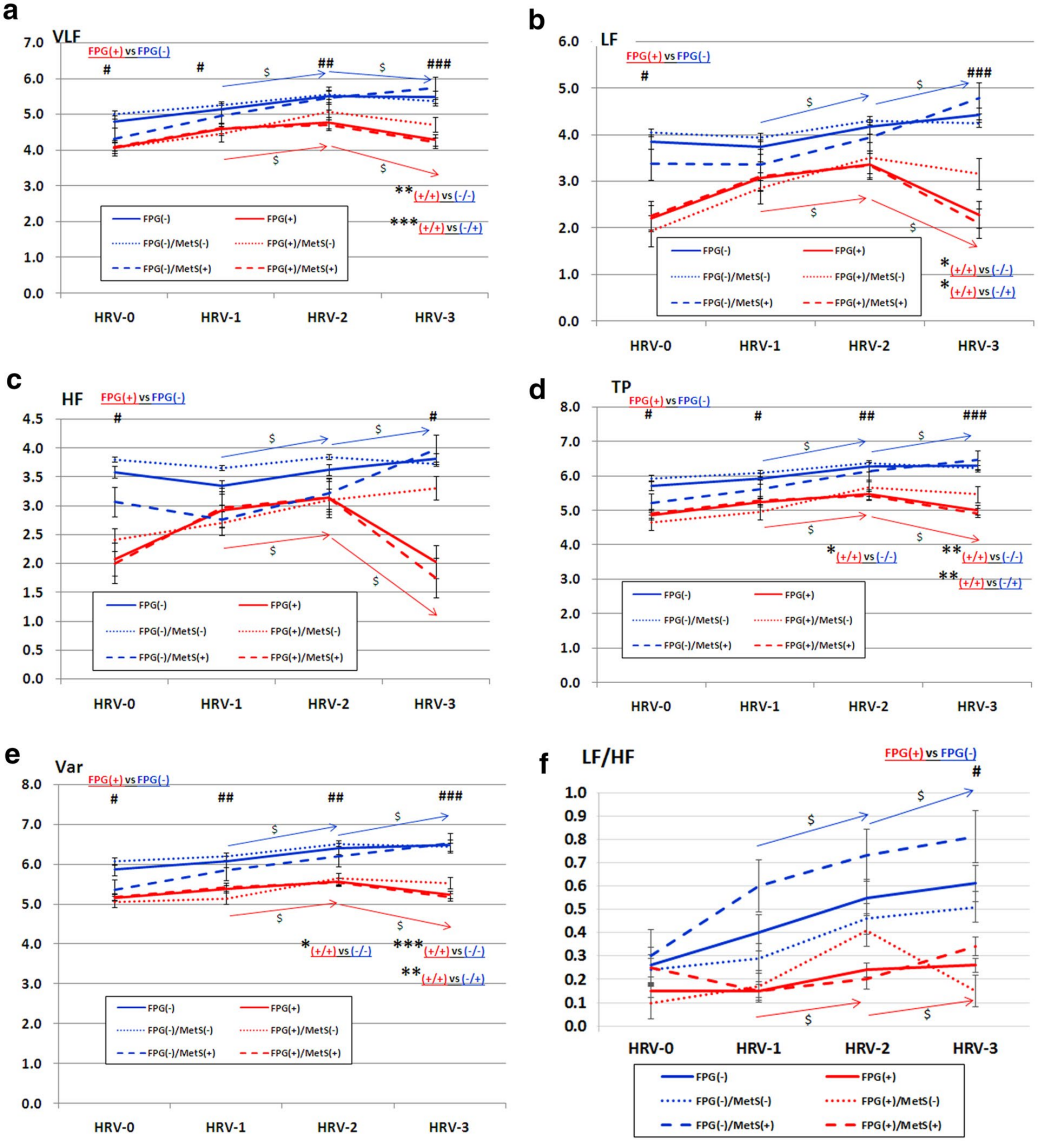
Plots comparing heart rate variability indices during hemodialysis among the four groups. **a** VLF, **b** LF, **c** HF, **d** TP, **e** Var, **f** LF/HF. *red solid line* FPG(+), n =  65; *blue solid line* FPG(-), n = 110; *red dashed line* FPG(+)/MetS(+), n = 54; *red dotted line* FPG(+)/MetS(-), n = 11; *blue dashed line* FPG(-)/MetS(+), n = 37; *blue dotted line* FPG(-)/MetS(-), n = 73. HRV-1, -2, and -3 were HRV measured at initial, middle, and late phase of the HD session, respectively. *#, ##, ###* denote p ≤ 0.05, ≤0.01, ≤0.001, respectively, between FPG(+) and FGP(-) groups. *, **, *** denote p ≤ 0.05, ≤0.01, ≤0.001, respectively, between two subgroups. *Blue* and *red arrow lines* respectively denote the trend of serial change of HRV in FPG(-) and FPG(+) groups. ^$^ denotes p ≤ 0.001. Units: Ln(ms2) in VLF, LF, HF, TP, and Var; Ln(ratio) in LF/HF ratio. *FPG* fasting plasma glucose, *HF* high frequency; *HRV* heart rate variability, *LF* low frequency, *MetS* metabolic syndrome, *TP* total power, *Var* variance of the R-R intervals, *VLF* very low frequency

The proportion of MetS(+) in the patients with FPG(+) (54 out of 65 patients, 83.1 %) was significant higher than that in FPG(−) group (37 out of 110 patients, 33.6 %) (p < 0.001). Then we further used MetS(+/−) to subcategorize patients in FPG(+) and FPG(−) groups and grouped them into four groups, namely, FPG(+)/MetS(+) (n = 54), FPG(+)/MetS(−) (n = 11), FPG(−)/MetS(+) (n = 37), and FPG(−)/MetS(−) (n = 73). Compared with FPG(−)/MetS(−) group, the patients in the FPG(+)/MetS(+) group had lower VLF-2, TP-2, Var-2, VLF-3, LF-3, TP-3, and Var-3. Moreover, the patients in the FPG(+)/MetS(+) group had lower VLF-3, LF-3, TP-3, and Var-3 comparing with the FPG(−)/MetS(+) group (Table 3; Fig. 1).

### Subgroup analysis in patients with/without diabetes mellitus

To further address the role of DM in affecting HRV indices in current study, we performed a subgroup analysis categorizing patients by the presence or absence of DM. In the DM(+) subgroup (n = 65), none HRV indices were significantly different between MetS(+) and MetS(−) groups, while TG was the only components of MetS associated with differences of HRV indices. The TG(+) group had significantly lower VLF-0 (2.92 ± 1.44 versus 4.68 ± 1.82, p = 0.008), TP-0 (3.54 ± 1.60 versus 5.36 ± 1.99, p = 0.013), Var-0 (3.93 ± 1.40 versus 5.63 ± 1.95, p = 0.014), but higher LF/HF ratio-0 (0.96 ± 1.23 versus 0.13 ± 1.02, p = 0.049) and LF/HF ratio-1 (0.56 ± 1.14 versus 0.13 ± 1.15, p = 0.031) than the TG(−) group.

In the DM(−) subgroup (n = 110), MetS(+) and MetS(−) were also not associated with differences in any HRV indices. The only two components with significant impacts on HRV indices were WC(+) which was associated with significant lower VLF-0 (4.23 ± 1.79 versus 5.15 ± 1.33, p = 0.017) comparing with WC(−) patients, and FPG(+) which was associated with lower LF/HF ratio-0 (0.10 ± 1.15 versus 0.26 ± 1.14, p = 0.003) than FPG(−) group.

From current study enrolling all 175 patients, we found that the existence of MetS had some impacts on HRV at varied phases including before and during HD. Among the five components, FPG(+) played a significant and probably the major role on the influences of MetS. In the subsequent subgroup analysis, TG(+) showed its impact on HRV indices in DM(+) subgroup, while WC(+) and FPG(+) were exhibited to have influence on HRV in DM(−) subgroup. It’s worthwhile to mention that the extend of influence of FPG(+) on HRV was obviously decreased in the subgroup analyses. We considered this finding a bias from the imbalanced population distribution because the proportion of FPG(+):FPG(−) in the DM(+) (n = 55) and DM(−) (n = 120) subgroups were 100:0 and 9.1:90.9, respectively.

## Discussion

To the best of our knowledge, current study is the first one to investigate the impact of MetS on HRV during HD in the chronic uremic population. And we demonstrated the serial changes of HRV indices during HD, which represented autonomic compensation under stress. The study has several main findings. First, the impact of FPG(+) and/or MetS(+) outstood the influence of uremic autonomic dysfunction. Second, FPG criterion was the most important component of MetS and carried the most powerful influence on cardiac ANS. Its impact was even higher than that of MetS. Third, the HRV indices of the FPG(−) group increased continuously throughout the whole HD process, while that of the FPG(+) group increased initially then decrease dramatically at a later phase of HD.

### Impact on ANS: from diseased kidney

To maintain human vital functions, the ANS has to promptly respond to various stimuli [26]. However, sympathetic overactivity which may be caused by diseased kidney contributes to the progression of heart and kidney diseases [27]. The sympathetic activity increases gradually accompanying the deterioration of renal function since early stage of renal dysfunction [28]. Nevertheless, the sympathetic activity trends to decrease in patients who underwent HD for a longer period and it suggests that sympathetic nervous functions might be affected by the duration of HD [29].

The HRV indices in patients with chronic kidney disease are lower than healthy individuals [30], and diminished HRV indices are indicative of cardiac ANS impairment and subsequent development of chronic kidney disease [31]. In uremic patients on maintenance HD, the increased LF/HF ratio with low values of both LF and HF is suggestive of shift of the cardiac ANS balance toward sympathetic predominance [27]. In the aspect of clinical intervention, aerobic training are found to increase HRV and cardiac vagal tone in both healthy and illed individuals [32].

### Impact on HRV: from MetS and its components

Current study found that the baseline values (HRV-0) of almost all HRV indices (except LF/HF ratio) were significantly lower in the patients with FPG(+) (also known as impaired fasting glucose (IFG)) than those with FPG(−). As to the serial measurement of HRV during HD, some indices (VLF, TP, and Var) were of significantly lower values throughout the whole HD session in FPG(+) group, but other indices (LF, HF, and LF/HF ratio) only showed the difference at late phase of HD. HD-related HRV disturbance, such as hemodynamic stress or electrolyte level alteration, might contribute to the absence of the difference between FPG(+) and FPG(−) groups at earlier HD phase.

The relationships among ANS function, DM and cardiovascular diseases have been addressed in several articles [33–35]. Both sympathetic and parasympathetic activity are documented to link to insulin resistance and type 2 DM [36, 37], suggesting the critical role of abnormal glucose metabolism on autonomic dysfunction. Even IFG, a milder form of glucose metabolism disturbance, was found to be associated with decreased HRV values and considered as an independent predictor for cardiovascular disease mortality in non-uremic patients after adjustment with other traditional cardiovascular risk factors [38]. The findings in current study consisted with the above-mentioned knowledge, and further emphasized that the impact of FPG(+) on cardiac ANS still pronounced even in the presence of uremic autonomic dysfunction.

Stuckey et al. [22] reviewed 14 investigations evaluating the relationship between HRV and MetS in non-uremic population, and found that IFG might be associated with decreased LF and HF, increased LF/HF ratio, along with neural effects on TP and VLF. The impact of IFG could be roughly interpreted as decreasing the parasympathetic tone and the mixture of both sympathetic and parasympathetic tone, but not yet reaching the decrease of total autonomic tone. However, the results were not totally the same with our findings in which both sympathetic and parasympathetic tone, as well as total autonomic nervous tone were decreased in FPG(+) patients by means of significantly decreased values of VLF, LF, HF, TP, and Var. The influence of uremic autonomic dysfunction may be responsible for the diverse findings between current study and others.

Among non-uremic population, the HRV values were of significant differences in time domain measures with presence of ≧1 components, and in frequency domain measures with presence of ≧3 components of MetS [21]. And the individual components of MetS played certain roles in affecting HRV [22]. Nonetheless, among the uremic patients in current study, only FPG(+) carried significant impact on HRV, while the rest four components of MetS including WC, TG, HDL, and BP showed only little influences on HRV (Table 2). The influence of FPG(+) was even higher than the impact of MetS. In the analyses among four groups categorized by FPG(+/−) and MetS(+/−), patients with FPG(+) were likely to have lower HRV than those with FPG(−), regardless presence or absence of MetS (Table 3; Fig. 1). Two possible explanations for the differences of influence on HRV between non-uremic and uremic patients were proposed. First, the impacts on HRV from the four components might be masked by the uremia-associated situation including uremic autonomic neuropathy. The BP issue is complicated because it reflects not only ANS activity but also fluid status in uremic patients. The influence of lipid profiles on patient outcomes, and the recommendation for lipid management in uremic population is different from general population [39]. Besides, WC or obesity may only play a modest role in affecting ANS activity because in women with polycystic ovary syndrome, a entity with insulin resistance and sympathetic activation, the ANS activation is independent of metabolic disturbances and obesity [40]. And visceral adiposity index may provide a better predictive value for cardiovascular outcomes than WC in HD patients [41]. The second explanation might be illustrated by “reverse epidemiology phenomenon” in uremic patients. The phenomenon refers to that the traditional cardiovascular risk factors, such as obesity, high BP, and dyslipidemia, turn to play protective roles of cardiovascular system and result in lower mortality rates in uremic patients [42].

### The serial change of HRV indices during HD: the impact from FPG

No matter representing sympathetic, parasympathetic, or total autonomic activities, all HRV indices were of lower mean values indicating lower ANS activities throughout the entire HD session in FPG(+) patients than in FPG(−) group in current study. Besides, different from the HRV measures of FPG(−) group which increased throughout the HD process, the HRV in FPG(+) group tended to increase initially when the patients facing stress (HD with ultrafiltration), but decrease in the later phase of HD when the stress increased gradually. These findings echoed the known knowledge that DM is a risk factor of intradialytic hypotension [25, 43], of which one of the potential mechanisms is the incapability of increasing both sympathetic and parasympathetic activity in response to the stimulus during HD [5].

In our previous work [44] evaluating the association between intradialytic hypotension and HRV indices in uremic patients, we found that the values of most HRV indices were consistently lower throughout the whole HD course in the patients with intradialytic hypotension than those with intradialytic hypertension. And HRV indices were proved as independent predictors for intradialytic hypotension. Interestingly and meaningfully, the plots comparing the serial changes of most HRV indices during HD process between FPG(+) and FPG(−) groups in current study, were extremely similar with the plots comparing patients with intradialytic hypotension and intradialytic hypertension in previous study [44]. Taken these two studies together, the results exhibited the pathophysiology and mechanism of the axis from glucose metabolic abnormality, through ANS disturbance, to resulting in intradialytic BP change.

### Limitations

The current study has some limitations. First, HRV indices may be affected by dysrhythmia and some anti-hypertensive agents such as beta-blockers, angiotensin converting enzyme inhibitors, or angiotensin II receptor blockers. We had excluded patients with dysrhythmia at enrollment, but we didn’t exclude patients taking these anti- hypertensive agents due to the restriction of case numbers. However, the percentage of these drugs usage is similar in the two groups (Table 1). Second, the HRV indices were only measured in the index session of HD. The bias of sampling could not be excluded. Third, we only measured short-term HRV at baseline and three times at initial, middle, and late phases in the index HD. Information of 24-h long-term HRV are lacking. Fourth, the sympathetic tone in our patients was not evaluated by some direct methods such as recording muscle sympathetic nerve activity or checking plasma norepinephrine levels. Nevertheless, these direct methods are invasive and less practically available, and their predictive values have yet to be determined [27]. Fifth, the FPG criterion didn’t exclude patients with DM in current study. We found that FPG(+) had significant impact on most HRV indices throughout entire HD process. But we could not further address the impact of varied glucose metabolism abnormalities due to the bias from the imbalanced population distribution in current study. Further prospective study designed to compare the impacts on HRV during HD of patients with DM(+), FPG(+)/DM(−), and FPG(−) is recommended.

## Conclusions

In conclusion, the impact of FPG(+) and/or MetS(+) outstood the influence of uremic autonomic dysfunction, and FPG criterion was of the highest impact on HRV among the components of MetS in uremic patients. These results underscored the importance of interpretation and management for abnormal glucose metabolism.

## Abbreviations

ANOVA: analysis of variance
ANS: autonomic nervous system
BP: blood pressure
DM: diabetes mellitus
FPG: fasting plasma glucose
HD: hemodialysis
HDL: high-density lipoprotein
HF: high-frequency
HRV: heart rate variability
IFG: impaired fasting glucose
i-PTH: intact parathyroid hormone
LF: low-frequency
MetS: metabolic syndrome
TG: triglycerides
TP: total power
Var: variance
VLF: very low frequency
WC: waist circumference
