# Erratum to: Impact of metabolic syndrome and its components on heart rate variability during hemodialysis: a cross-sectional study

**DOI:** 10.1186/s12933-017-0562-2

**Published:** 2017-07-14

**Authors:** Yu-Ming Chang, Chih-Chung Shiao, Ya-Ting Huang, I-Ling Chen, Chuan-Lan Yang, Show-Chin Leu, Hung-Li Su, Jsun-Liang Kao, Shih-Ching Tsai, Rong-Na Jhen, Ching-Cherng Uen, On behalf of SMCKD (Saint Mary’s hospital Chronic Kidney Disease) study group

**Affiliations:** 1 Division of Nephrology, Department of Internal Medicine, Saint Mary’s Hospital Luodong, No. 160 Chong-Cheng South Road, Loudong, 265 Yilan Taiwan, ROC; 2 Division of Neurology, Department of Internal Medicine, Saint Mary’s Hospital Luodong, No. 160 Chong-Cheng South Road, Loudong, 265 Yilan Taiwan, ROC; 3 Department of Nursing, Saint Mary’s Hospital Luodong, No. 160 Chong-Cheng South Road, Loudong, 265 Yilan Taiwan, ROC; 4 Saint Mary’s Junior College of Medicine, Nursing and Management, No. 100, Ln. 265, Sec. 2, Sanxing Rd., Sanxing Township, 266 Yilan County Taiwan, ROC; 5 Graduate Institute of Clinical Medical Sciences, Chang Gung University, No. 259, Wenhua 1st Rd., Guishan Dist., Taoyuan, 33302 Taiwan, ROC

## Erratum to: Cardiovasc Diabetol (2016) 15:16 DOI 10.1186/s12933-016-0328-2

Since publication of the original article [1], affiliation 4 has changed from “Saint Mary’s Medicine, Nursing and Management College. No. 100, Ln. 265, Sec. 2, Sanxing Rd., Sanxing Township, Yilan County 266, Taiwan, ROC.” to “Saint Mary’s Junior College of Medicine, Nursing and Management. No. 100, Ln. 265, Sec. 2, Sanxing Rd., Sanxing Township, Yilan County 266, Taiwan, ROC.”
